# Identifying the impact of ARHGAP and MAP gene families on autism spectrum disorders

**DOI:** 10.1371/journal.pone.0306759

**Published:** 2024-11-08

**Authors:** Tianci Gao, Wenjun Dang, Zhimei Jiang, Yuwei Jiang

**Affiliations:** 1 College of Clinical Medicine, Jiamusi University, Jiamusi, HeilongJiang Province, China; 2 Jiamusi College, HeiLongJiang University of Chinese Medicine, Jiamusi, HeilongJiang Province, China; 3 College of Rehabilitation Medicine, Jiamusi University, Jiamusi, HeilongJiang Province, China; 4 Child Neurological Rehabilitation Key Laboratory of Heilongjiang Province, Jiamusi, Heilongjiang Province, China; Taipei Veterans General Hospital, TAIWAN

## Abstract

The rising incidence of Autism Spectrum Disorder (ASD) has become a major concern, affecting children’s psychological well-being and placing a significant strain on healthcare systems. Despite its impact, the etiological mechanisms underpinning ASD remain elusive. This study leveraged dorsolateral prefrontal cortex gene data from 452 individuals of European descent, sourced from the CommonMindConsortium, and examined ASD-related gene expression data from the Gene Expression Omnibus (GEO) database (GSE18123), along with Genome-Wide Association Studies (GWAS) data from the Lundbeck Foundation Integrated Psychiatric Research and Psychiatric Genomics Consortium. Expression quantitative trait loci data were sourced from the GTExv8 database. We employed Transcriptome-Wide Association Studies (TWAS) and Weighted Gene Co-expression Network Analysis (WGCNA) to pinpoint genes within ASD-associated susceptibility gene families (ARHGAP, MAP). Four genes—ARHGAP27, MAPT, ARHGAP19, and MAP1B—were scrutinized, and their biological implications were elucidated through Gene Ontology (GO) and Kyoto Encyclopedia of Genes and Genomes (KEGG) enrichment analyses. Protein-Protein Interaction (PPI) analysis and conditional analysis within the TWAS framework helped identify pivotal genes (ARHGAP27, MAPT). A subsequent verification phase involving Mendelian Randomization (MR) evaluated the potential causal links between the identified genes and ASD. The findings revealed no causal association between ARHGAP19, MAP1B, and ASD. In contrast, significant causal relationships were established for ARHGAP27 and MAPT, suggesting that ARHGAP27 may elevate ASD risk as a susceptibility gene, whereas MAPT appears to reduce the risk as a protective gene.

## Introduction

From a sociological viewpoint, humans are inherently social beings, and maintaining free and healthy social interactions is essential for mental health. Autism Spectrum Disorder (ASD) is characterized by the presence of restrictive behaviors, repetitive patterns of behavior, social challenges, language deficits, specialized interests, and an acute sensitivity to environmental changes, which often precludes the establishment of free and healthy social [1]. Consequently, elucidating the etiology and pathogenesis of ASD is of paramount importance, potentially mitigating the psychological burden and reducing the risk of secondary complications such as anxiety, depression, and suicidal tendencies among affected individuals [2]. While social, familial, and behavioral factors have been implicated in the onset of ASD, genetic contributions are increasingly recognized as significant. For instance, monozygotic twins demonstrate a 60% [3] concordance rate for classic autism, and genome-wide association studies have pinpointed specific single nucleotide polymorphisms(SNP) implicated in ASD [4]. Nonetheless, the functional roles of these SNPs in the biological and biochemical [5] pathways pertaining to autism remain elusive, posing a barrier to a comprehensive understanding of the disorder’s underlying mechanisms. Genes are fundamental to the genetic blueprint and influence the development, function, and traits of organisms. Despite advances, there is sparse knowledge regarding the genes within the central nervous system that contribute to ASD. Research has shown that certain genes in the ARHGAP and MAP families are noteworthy for their potential correlations with ASD. The ARHGAP family significantly influences neuronal integrity by modulating Rho GTPases activity. Notably, gene mutations such as those in ARHGAP11B have been identified in ASD patients, indicating its conceivable involvement in ASD etiology [6]. Concurrently, the MAP family is instrumental in maintaining neuronal structure and microtubule dynamics, both essential for proper neuronal development and functioning. In individuals with ASD, atypical expressions of MAP1A and MAP2, which can disrupt intraneuronal and extraneuronal signal transmission, have been implicated in compromising neural network operations [7, 8]. This article intends to explore the vulnerability of certain genes within the Rho GTPase activating protein (ARHGAP) [9, 10] and Microtubule-Associated Protein (MAP) [11] gene families to ASD. Our methodology includes integrating Expression quantitative trait loci (eQTL) [12] datasets from the human brain and Genome-Wide Association Studies (GWAS) statistics pertinent to ASD to conduct Transcriptome-Wide Association Studies TWAS [13], aiming to discern genes susceptible to ASD. Subsequently, using Weighted Gene Co-expression Network Analysis (WGCNA), we examined the gene expression profiles in the peripheral blood of autism patients to pinpoint the core genes implicated in ASD [14]. Additionally, we scrutinize GWAS data concerning ARHGAP and MAP family genes identified through these techniques. Subsequent to this, Mendelian Randomization (MR) [15] is employed to substantiate the susceptibility of genes within the ARHGAP and MAP families to ASD. Complementary to this, Protein-Protein Interaction (PPI) analyses [16] and conditional TWAS assessments serve to pinpoint core genes, while Gene Ontology (GO) [17] and Kyoto Encyclopedia of Genes and Genomes (KEGG) [18] enrichment analyses provide insight into the biological relevance of these genes in the discovery phase.

## Materials and methods

### Ethics approval and consent to participate

I would like to confirm that the data used in this study is publicly available and can be reused without restrictions under an open license. All datasets in this research have been downloaded from public databases. These public databases permit researchers to download and analyze public datasets for scientific purposes, thereby negating the need for ethical approval.

**Fig 1:**
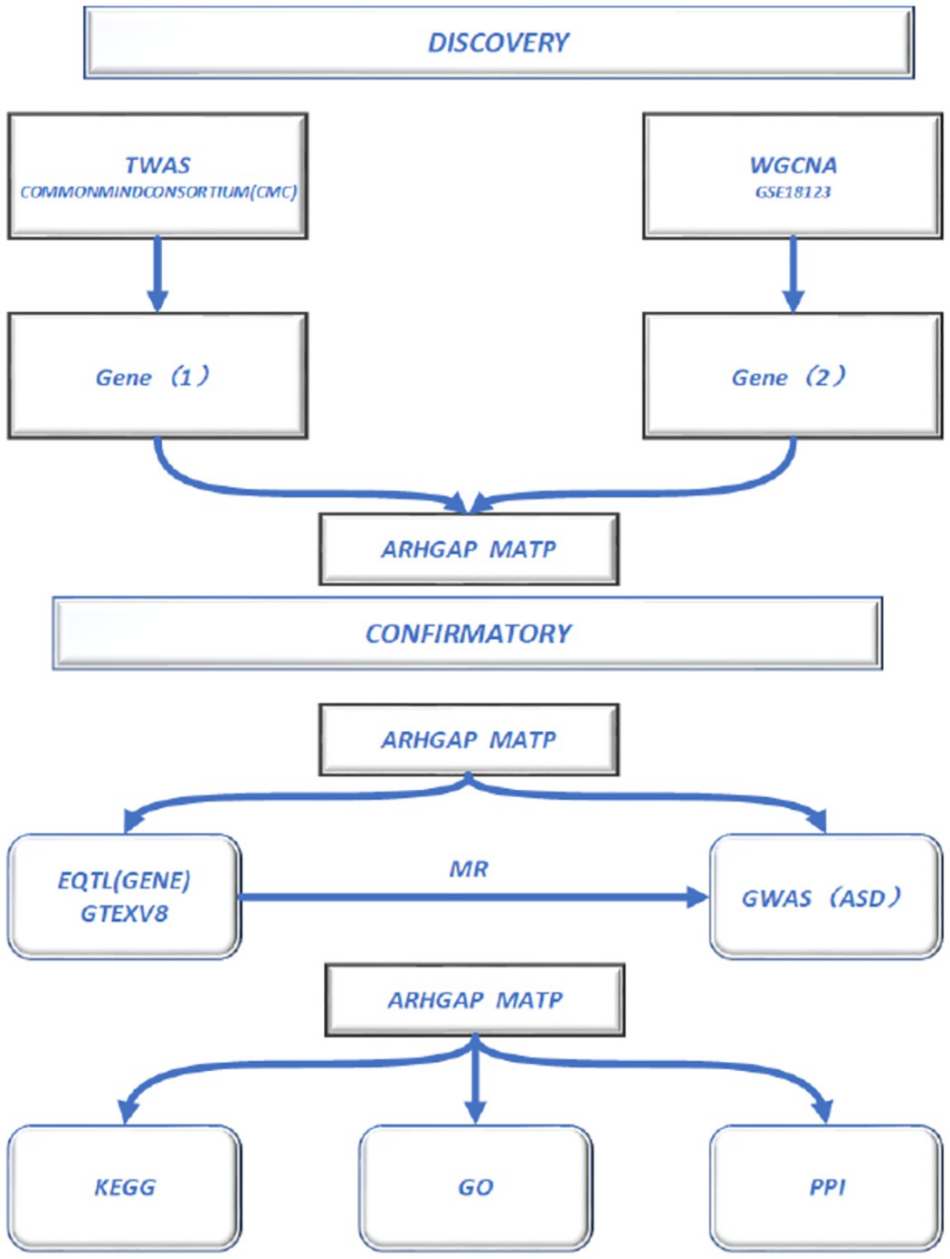
Study flowchart. ASD Autism Spectrum Disorder, TWAS Transcriptome-Wide Association Analysis, WGCNA Weighted Gene Co-Expression Network Analysis, eQTL Expression Quantitative Trait Loci, GO Gene Ontology, KEGG Kyoto Encyclopedia of Genes and Genomes, PPI Protein-Protein Interaction, MR Mendelian Randomization.

The flowchart of the study was presented in Fig 1.

### Dataset

The transcriptome reference genome used in the TWAS for the human brain is derived from dorsolateral prefrontal cortex (dlPFC) data of a research sample comprising 452 Europeans, provided by the CommonMind Consortium (CMC). It was found that expressions of 5,420 mRNAs are heritable. These mRNA weights are available for query and retrieval on the Gusev Laboratory’s FUSION project website (http://gusevlab.org/projects/fusion/), after which we deleted any missing values, as well as the mRNA types for which detailed information was not identifiable, along with non-heritable information. The peripheral blood gene expression dataset for ASD patients, GSE18123, is sourced from the GEO database. It encompasses genetic data from 170 ASD patients and 115 control individuals, all collected from various hospitals in the Boston area. We utilized the Perl scripting language (version 5.30) to categorize the gene expression and sample information for both the case group and the control group. We also transformed and completed the information for gene names and their expression levels for each category. Data retrieval is available at https://www.ncbi.nlm.nih.gov/. In the MR analysis for ASD, data from the GWAS were obtained from the meta-analysis results file. This file was collaboratively released by the Lundbeck Foundation Initiative for Integrative Psychiatric Research (iPSYCH) and the Psychiatric Genomics Consortium (PGC) in November 2017. The study comprised a total sample size of 46,351 participants, with 18,382 diagnosed cases and 27,969 controls." [19]. The comprehensive results are available after obtaining the necessary authorization from the PGC website (https://ww.med.unc.edu/pgc). The original study encapsulates data from 46,351 individuals of European ancestry and comprises 8,643,988 SNPs. Susceptibility gene data for ASD were retrieved from the GTExv8 [20] database, designed to facilitate research into the connections between gene expression and genetic variation in humans. This dataset includes extensive genotyping of donors and evaluates genetic variations within their genomes. Using brain tissue eQTL data as an instrumental variable, the study selected variants with a *p-value <1e*-5 [21]. This data can be downloaded from the GTEx portal website(https://www.gtexportal.org/home/).

### Statistical analyses

#### TWAS

The FUSION approach is a robust analytic framework that integrates gene expression with GWAS summary statistics to identify genes associated with complex traits via cis-regulatory expressions [22]. We executed the FUSION methodology as per the original authors’ instructions and settings, with the resultant visual analyses performed in R software version 4.3.2.

#### WGCNA

Using the "WGCNA" package in R, we constructed an unweighted gene co-expression network. Initial steps involved remedying missing data and executing cluster analysis on the sample set. A soft-thresholding power (β) was determined based on scale-free topology criteria to erect a biologically meaningful network. This was followed by constructing a topological overlap matrix (TOM) from the adjacency matrix and identifying gene modules with the dynamic tree-cut algorithm. We also computed gene significance (GS) and module membership (MM) [23] for delineation of associations with clinical traits. A feature gene network visualization assisted in hypothesizing potential ASD-related gene targets.

#### Functional enrichment

Functional enrichment assessments were performed on gene members of the ARHGAP and MAP families identified in previous stages. Such evaluations employed both GO and KEGG resources, with significant categories (*p < 0*.*05*) visualized using R software version 4.3.2.

### Construction of the Protein-Protein Interaction (PPI) network

Subsequently, using STRING (https://cn.stringdb.org/), a PPI network was constructed for ARHGAP and MAP gene families to evaluate gene interconnectivity. The network parameters were set for human organisms, with an interaction confidence score minimum of 0.15, and removal of disconnected nodes was performed. The PPI network data was exported into CSV format and imported into Cytoscape software (version 3.10.1), where the cytoHubba plugin calculated the critical core genes.

### MR validation

In MR analysis, SNPs used as instrumental variables must fulfill three prerequisites [24]: (1) robust association with the exposure factor, (2) independence from confounding variables, and (3) an association with clinical outcomes solely via the exposure factor [25]. To mitigate bias due to linkage disequilibrium, selected SNPs should have an r2 < 0.001 with a genetic distance exceeding 10,000 kb [26]. These variables were extracted from the GWAS dataset, including substantial data on effect alleles, non-effect alleles, effect sizes (β), standard errors (SE), and *p-values*.

## Results

### TWAS analysis results

**Fig 2:**
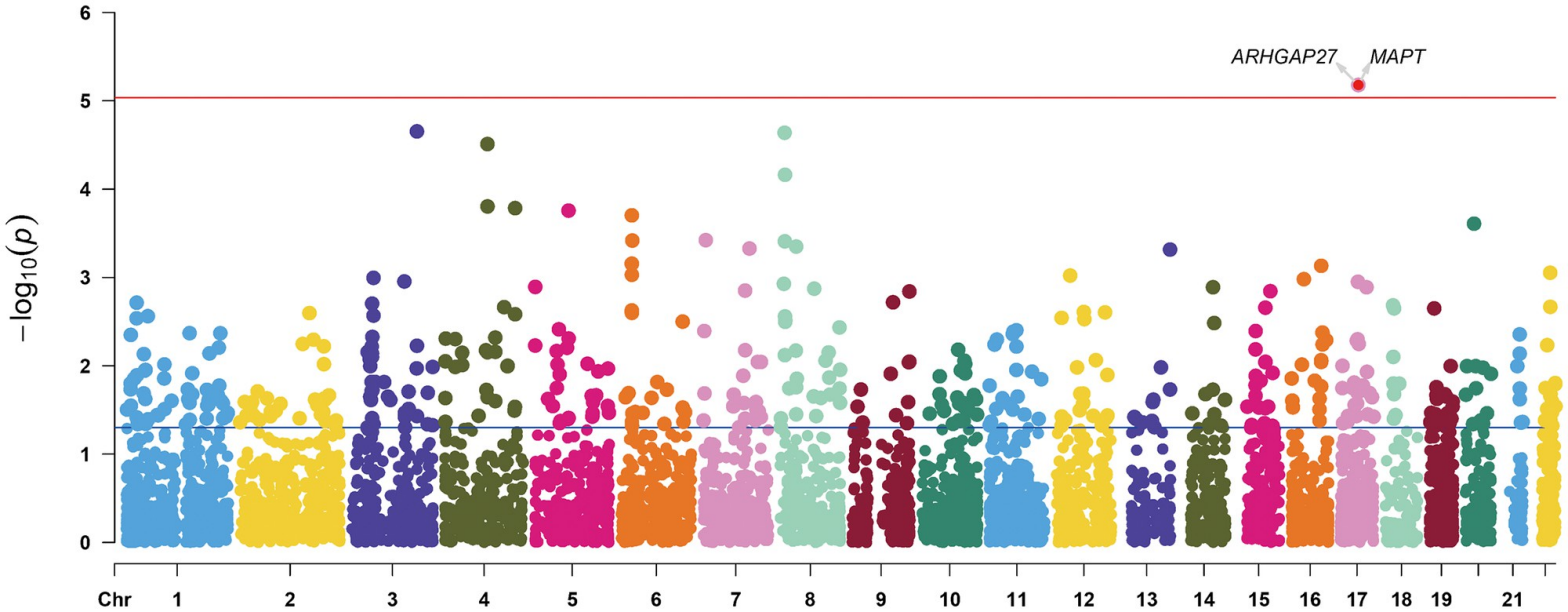
TWAS. A Manhattan plot for the ASD TWAS, integrating the ASD GWAS with dorsolateral prefrontal cortex samples from European individuals recruited by the CommonMind Consortium (N = 452). Each dot represents a single association test between a gene and ASD, ordered by genomic position on the x-axis and the association strength on the y-axis expressed as the -log10(P) of a z-score test. The discovery TWAS identified 2 expression quantitative trait loci associated with ASD, and the red horizontal line represents the significance threshold, set at the highest unadjusted p-value that is below the threshold (*p = 9 × 1e-5*).

The TWAS identified two genes with cis-regulatory expressions in brain tissues significantly associated with autism risk (refer to Table 1, Fig 2).

**Table 1:** In the TWAS analysis results, the genes within the top 20 p-values, ARHGAP27 and MAPT, reached the significance threshold.

PANEL	ID	CHR	P0	P1	TWAS.Z	TWAS.P	Tissue
CMC	ARHGAP27	17	43471267.00	43503012	4.5056	6.62E-06	CMC.BRAIN
CMC	MAPT	17	43971747.00	44105699	-4.5056	6.62E-06	CMC.BRAIN
CMC	MLF1	3	158288952.00	1.58E+08	4.24103	2.22E-05	CMC.BRAIN
CMC	PINX1	8	10622883.00	10697299	-4.2331	2.30E-05	CMC.BRAIN
CMC	MANBA	4	103552642.00	1.04E+08	-4.16699	3.09E-05	CMC.BRAIN
CMC	CTSB	8	11700033.00	11725646	3.9802	6.88E-05	CMC.BRAIN
CMC	CISD2	4	103790134.00	1.04E+08	-3.78039	0.000157	CMC.BRAIN
CMC	AADAT	4	170981372.00	1.71E+08	-3.76927	0.000164	CMC.BRAIN
CMC	ATG10	5	81267843.00	81551216	-3.75306	0.000175	CMC.BRAIN
CMC	HIST1H4C	6	26104175.00	26104565	3.7211	0.000198	CMC.BRAIN
CMC	PLK1S1	20	21106623.00	21227258	3.6663	0.000246	CMC.BRAIN
CMC	DAGLB	7	6448746.00	6523849	-3.55472	0.000378	CMC.BRAIN
CMC	ZNF204P	6	27325601.00	27343153	3.5521	0.000382	CMC.BRAIN
CMC	FAM167A	8	11278972.00	11324276	3.5469	0.00039	CMC.BRAIN
CMC	TM2D2	8	38846326.00	38854041	3.5109	0.000447	CMC.BRAIN
CMC	LOC401397	7	112756772.00	1.13E+08	3.49713	0.00047	CMC.BRAIN
CMC	DCUN1D2	13	114110133.00	1.14E+08	-3.4903	0.000483	CMC.BRAIN
CMC	HIST1H4B	6	26027123.00	26027480	3.39	0.000699	CMC.BRAIN
CMC	HIST1H2AB	6	26033319.00	26033796	3.39	0.000699	CMC.BRAIN

P0: The starting base pair position of a genetic variant on the chromosome.

P1: The ending base pair position of a genetic variant on the chromosome.

P1: The ending base pair position of a genetic variant on the chromosome.. TWAS.Z: The Z-score from a transcriptome-wide association study, indicating the strength and direction of the association.

**Fig 3:**
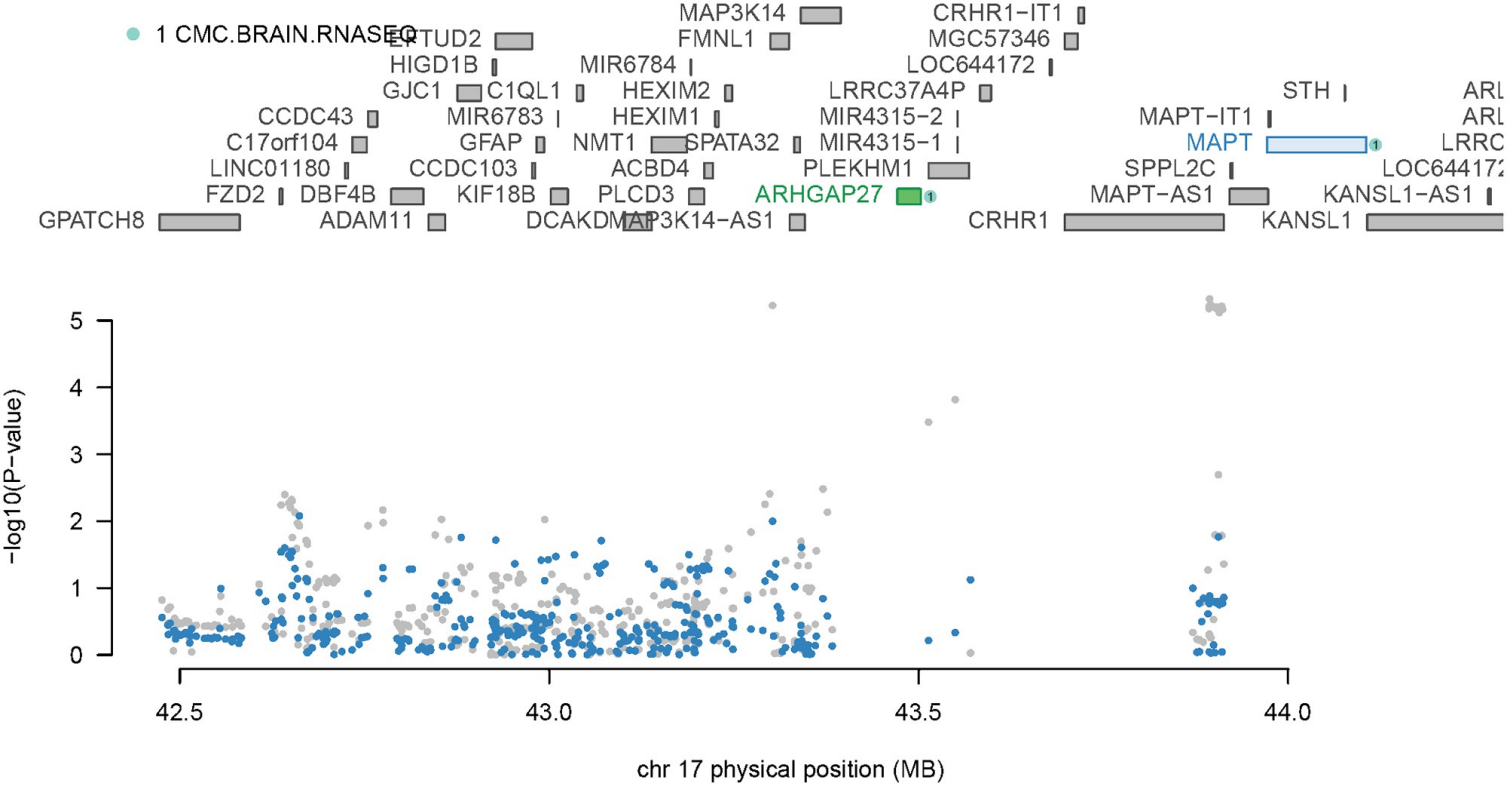
Joint conditional analysis in TWAS. Green represents joint genes, while blue indicates genes that reach the significance threshold (*p = 9 × 1e-5*) but only show marginal signals.

Among the evaluated genes, two are situated within a proximal distance on chromosome 17 (~468.7 kb apart). Through joint conditional TWAS analysis, we established the significant role of ARHGAP27 (p < 0.05), while MAPT did not retain significance after accounting for ARHGAP27 (see Fig 3).

### WGCNA analysis results

**Fig 4:**
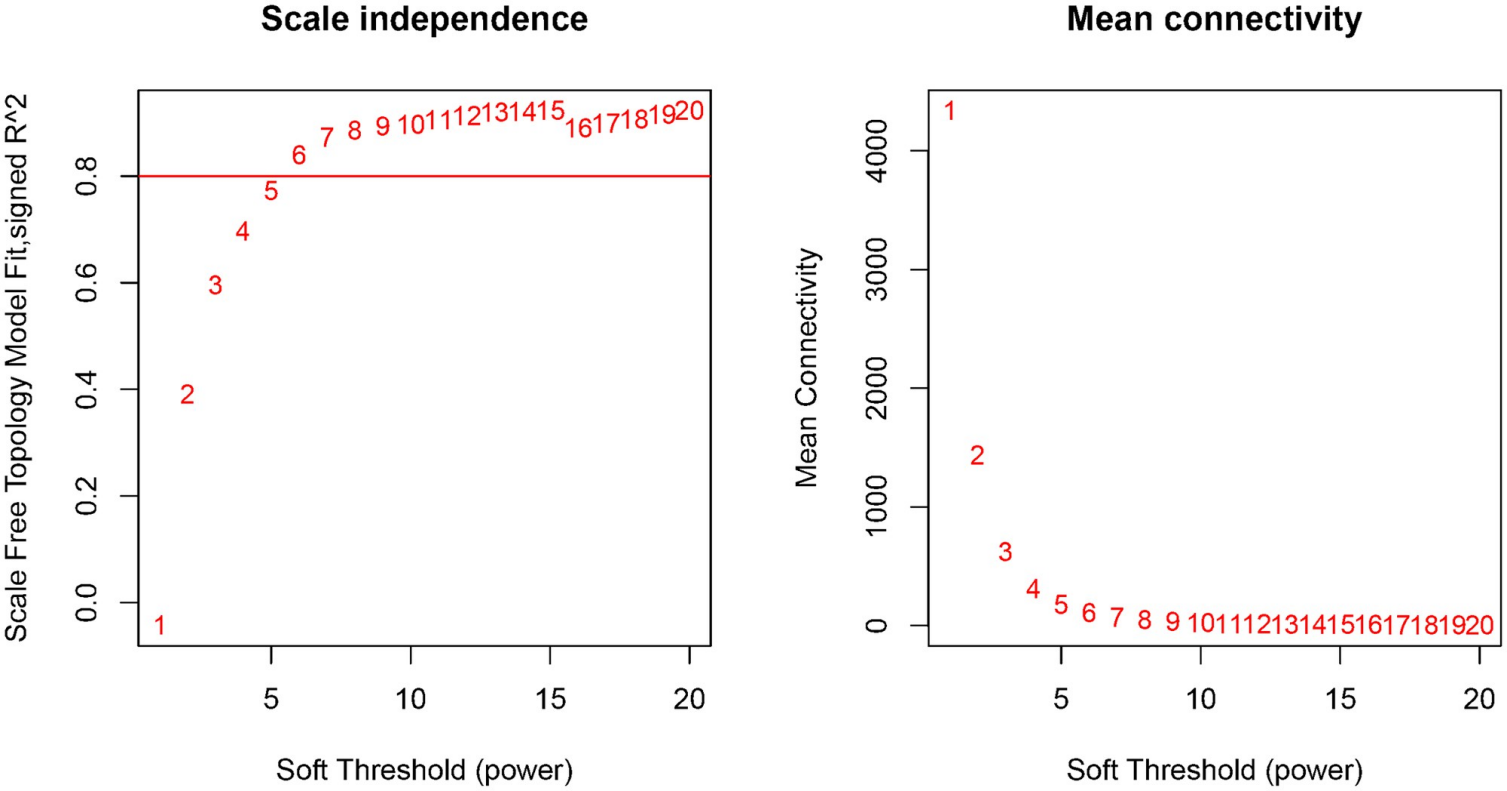
Selection of optimal soft threshold power for constructing gene co-expression networks. On the left, analysis of network topology for various soft thresholds. The X-axis represents the soft threshold power. The Y-axis represents the scale for the topology model fitting index. On the right, mean connectivity.

**Fig 5:**
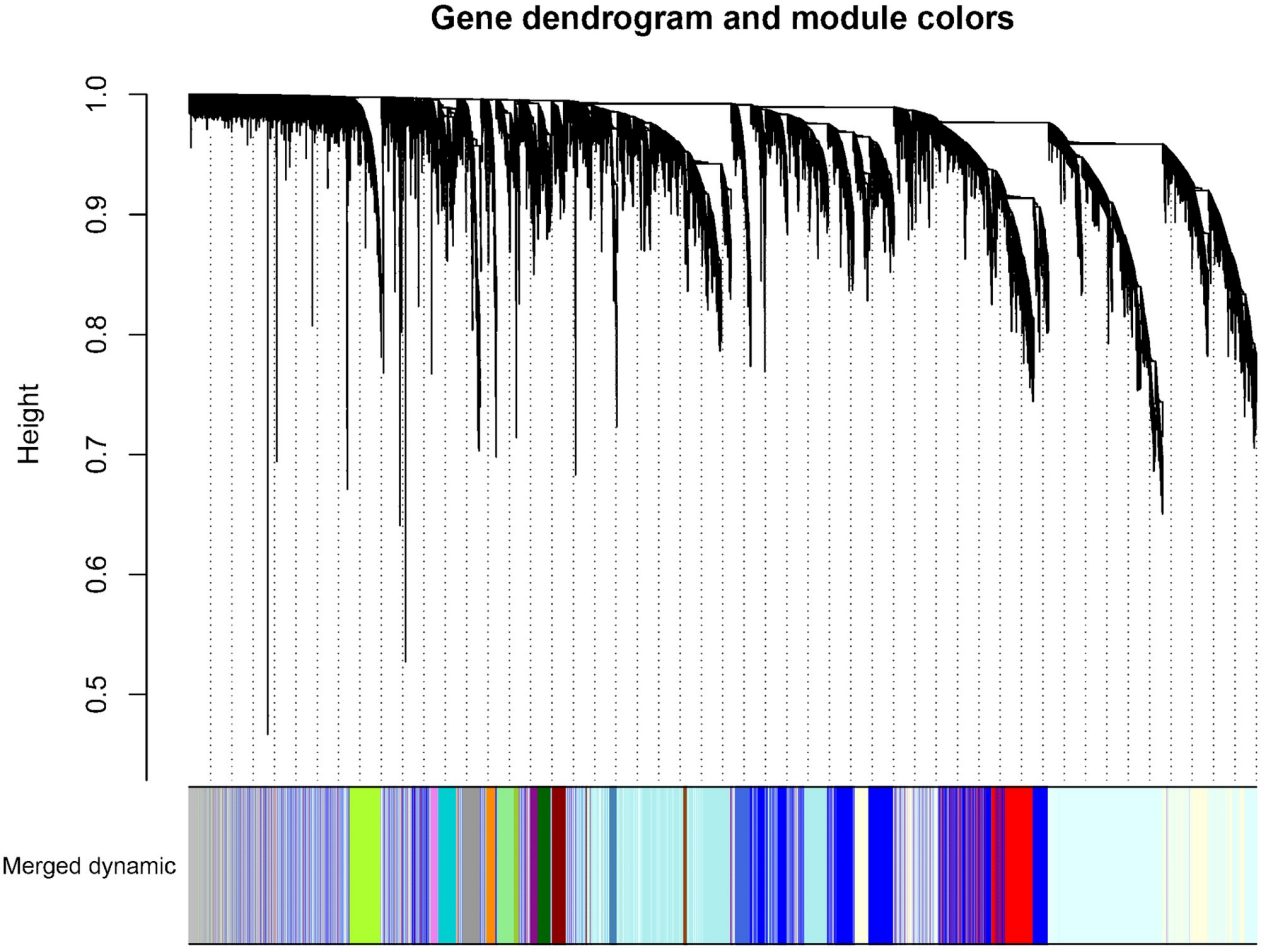
Identification of modules specifically associated with ASD. Cluster tree diagram and module feature relation diagram are used to divide modules.

**Fig 6:**
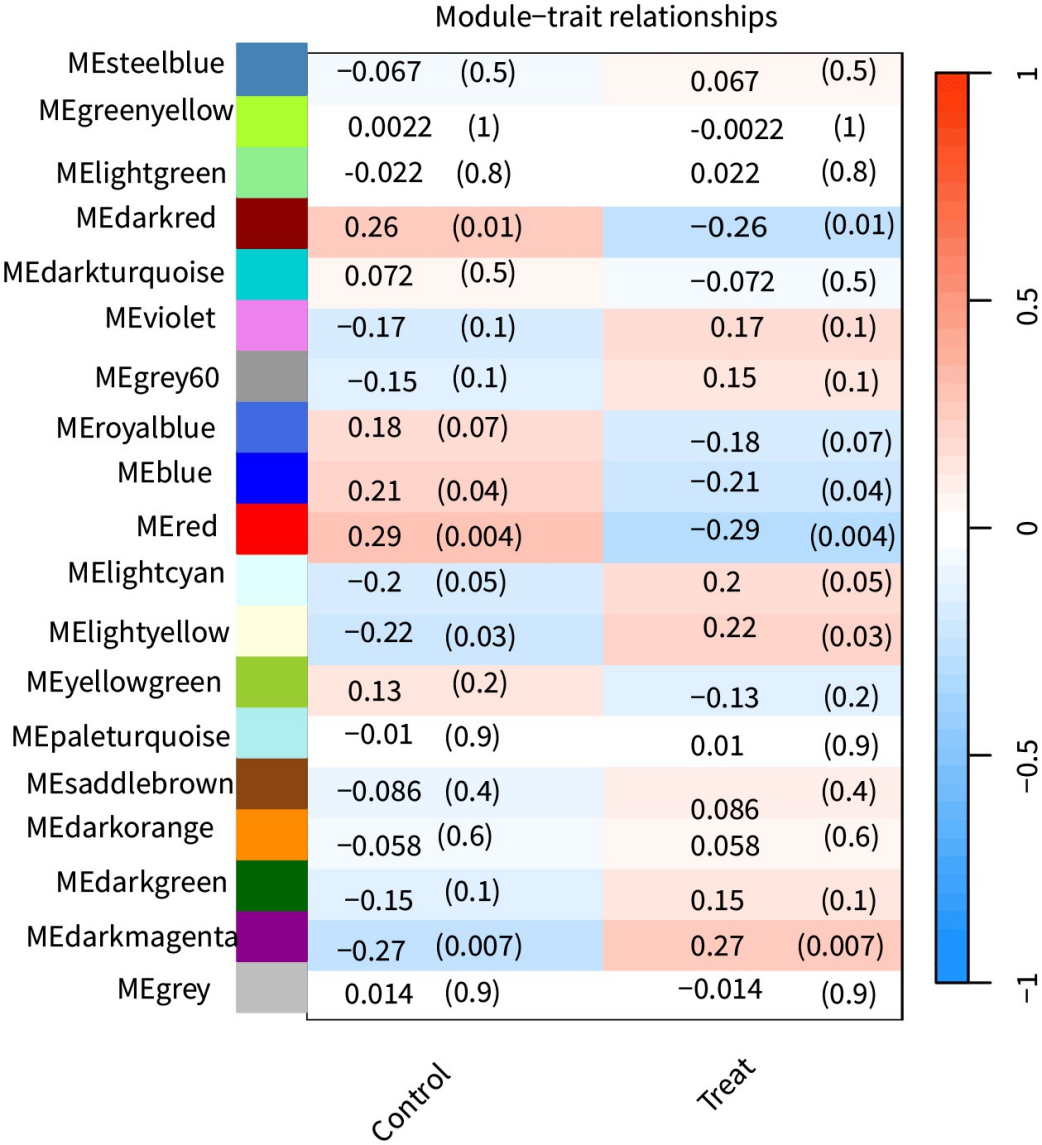
Shows the heatmap of module characteristic genes associated with ASD at different stages. The first value in each cell is the correlation coefficient; positive values indicate positive correlation, and negative values indicate negative correlation. The value in parentheses is the p-value; values less than 0.05 indicate significant correlations.

After performing WGCNA on the GSE18123 dataset, we identified several vital genes associated with ASD. By setting the soft-thresholding power to 6 (R^2 = 0.8), we constructed a scale-free network topology. Specifically, choosing a soft-thresholding power of around 6 allows the network to exhibit scale-free properties while maintaining reasonable connectivity, as shown in Fig 4. Through hierarchical clustering of the dendrogram, 19 ASD-related modules were automatically identified using the dynamic tree cut algorithm, as shown in Fig 6. The dynamic tree cut process is visualized in Fig 5. The correlations and p-values between these modules and clinical traits are detailed in Fig 6. Notably, the red module showed a significant correlation with late-onset ASD (r = -0.29, p = 0.004), marking it as the most clinically relevant module. The genes within this module were chosen for further in-depth analysis, and detailed results of the genes in this module can be found in S1 Table. These findings were also considered alongside results from the transcriptome-wide association study (TWAS).

### Functional enrichment analysis results

**Fig 7:**
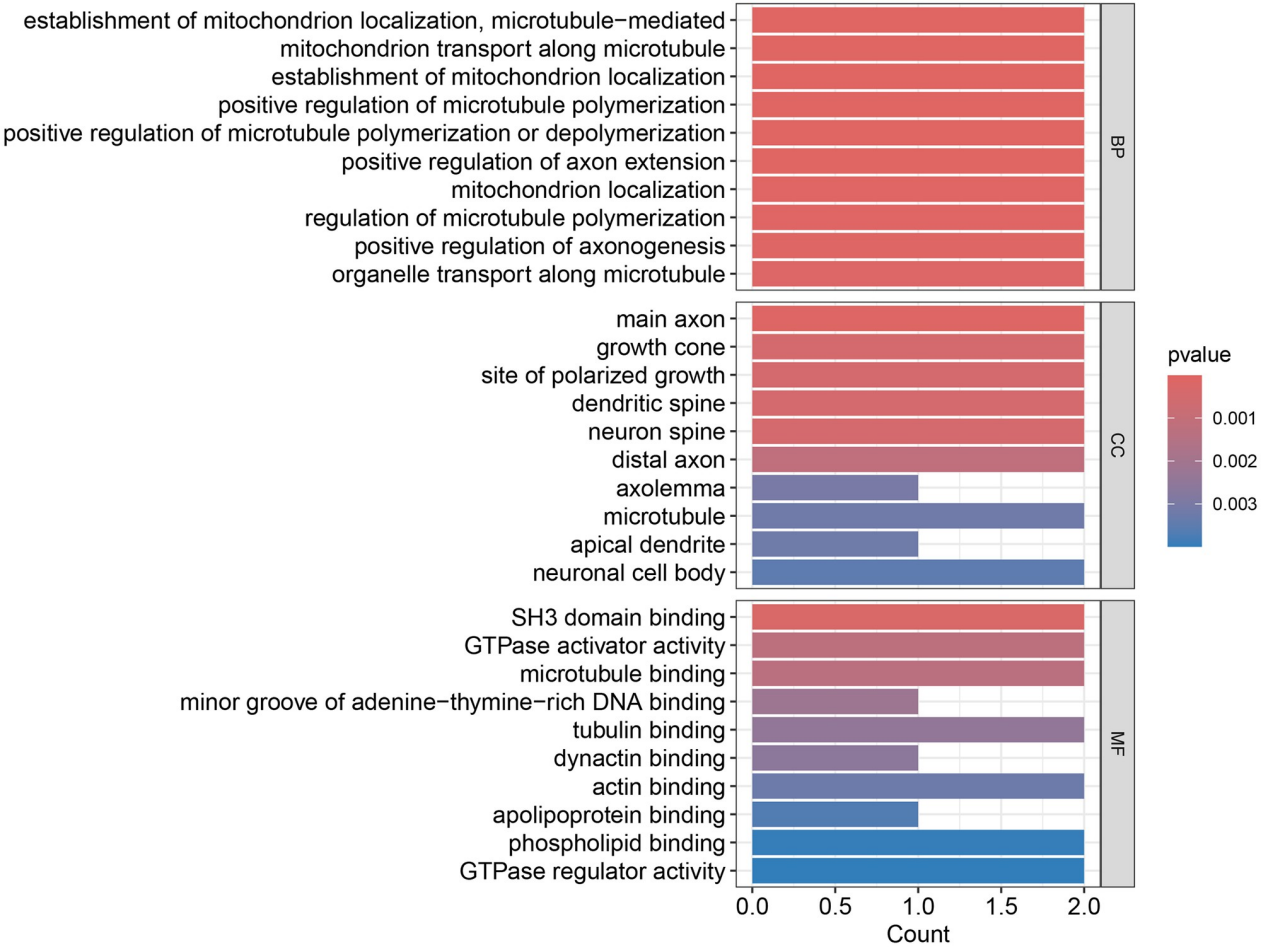
Shows the GO enrichment analysis performed on the ARHGAP27, MAPT, ARHGAP19, and MAP1B genes. Cellular component (CC), molecular function (MF), biological process (BP). When the p-value reaches 0.005, it indicates that the enriched genes are highly associated with ASD.

Integrating information from WGCNA and TWAS, four genes from the ARHGAP and MAPT gene families were identified for further study: ARHGAP27, MAPT, ARHGAP19, and MAP1B. Enrichment analyses through GO and KEGG in this discovery phase uncovered the diverse functional characteristics of these genes, with detailed results depicted in S2 Table and Fig 7 detailing the GO analysis results. In the classification of Biological Processes, functions included intracellular organelle transport, axonogenesis, and microtubule polymerization. Cellular Component analysis showcased their roles in neuronal structures such as dendrites and axons, and in the Molecular Function category, activities connected to various binding functions and GTPase regulation were highlighted. KEGG outcomes suggested a linkage to HIV-1.

### Analysis results of core gene members

**Fig 8:**
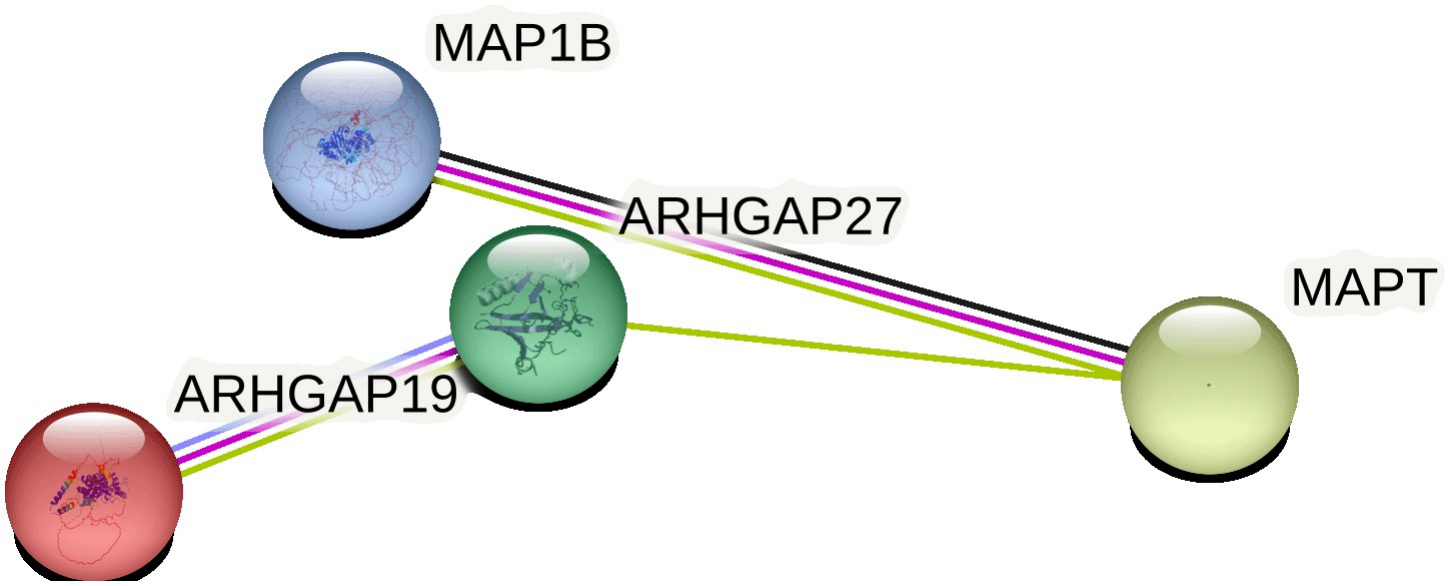
Protein-protein interaction networks are constructed with genes from the ARHGAP and MAPT family members, where the connections represent the interactive relationship between the proteins.

**Fig 9:**
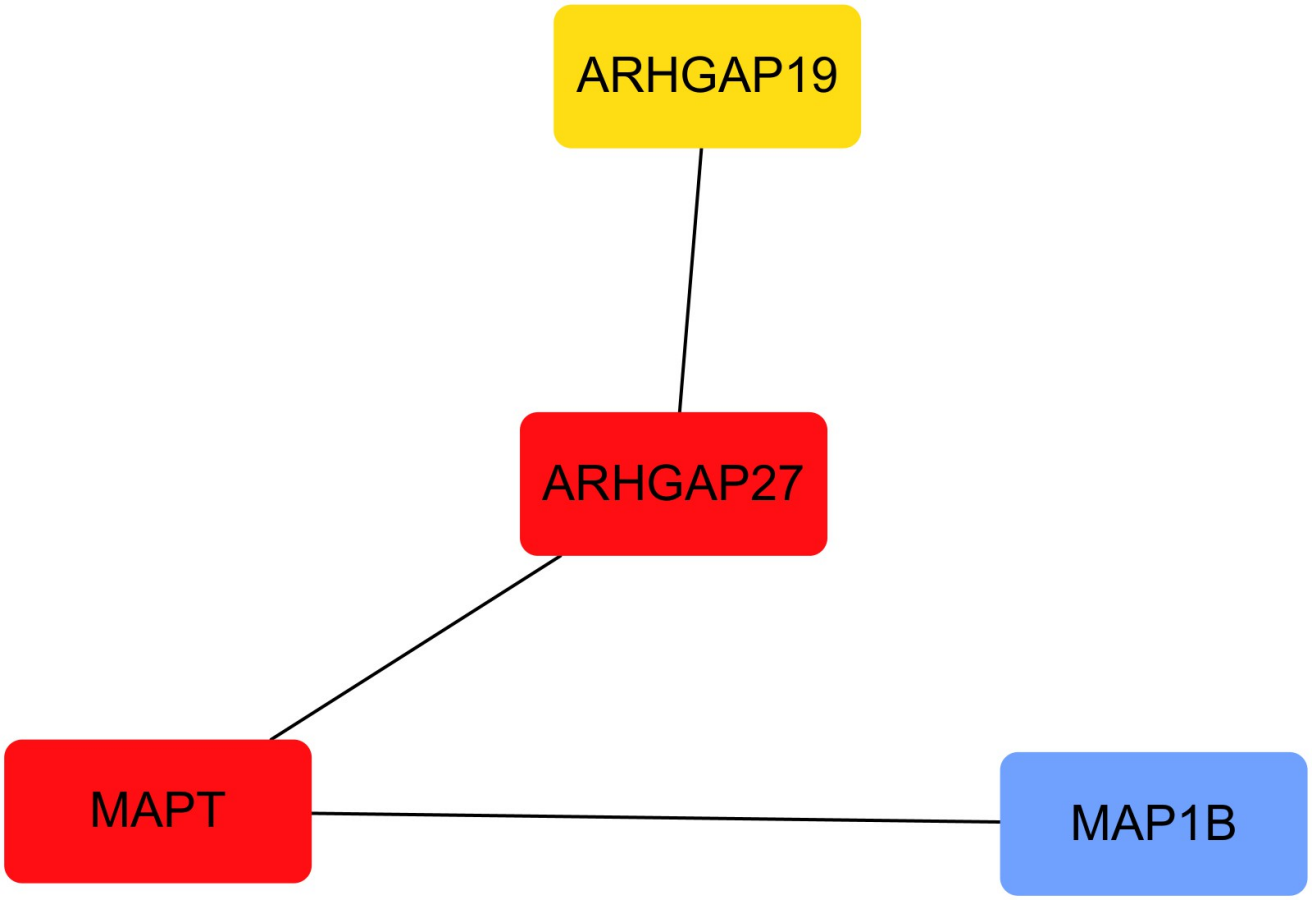
Core genes of the ARHGAP and MAPT family members are displayed. Those closer to red color represent genes referred to as the hub of the network.

Analysis Results of Core Gene Members The PPI network for ARHGAP27, MAPT, ARHGAP19, and MAP1B was illustrated in (Fig 8), where the cytoHubba plugin calculated centrality levels of the involved genes. ARHGAP27 emerged as the network’s core gene, denoted in (Fig 9).

### MR validation results

Subsequent MR analysis evaluated the putative causal relationships between the genes identified in the earlier phase and ASD (see Table 2 for detailed results).

**Table 2:** MR analysis results regarding gene family members show that OR > 1 indicates a risk factor, while OR < 1 indicates a protective factor.

Exposure	Outcome	Method	B	Se	Pval	Lo_ci	Up_ci	or	or_lci95	or_uci95	estimate
ARHGAP27	ASD	Wald ratio	0.17825	0.050315	0.000396	0.079632	0.276869	1.195124	1.082889	1.318993	1.20 (1.08–1.32)
MATP	ASD	Wald ratio	-0.18388	0.056844	0.001218	-0.29529	-0.07246	0.832038	0.744314	0.930101	0.83 (0.74–0.93)
ARHGAP19	ASD	Wald ratio	-0.03463	0.087453	0.692082	-0.20604	0.136774	0.965959	0.813798	1.146569	0.97 (0.81–1.15)
MAP1B	ASD	Wald ratio	-0.09528	0.057391	0.096889	-0.20776	0.01721	0.909122	0.812401	1.017359	0.91 (0.81–1.02)

Lo_ci and Up_ci represent the lower and upper bounds of the 95% confidence interval for the p-value, respectively. or_lci95 and or_uci95 represent the lower and upper limits of the 95% confidence interval for the odds ratio (OR), respectively.

This analysis confirmed a significant causal association for ARHGAP27 and MAPT with ASD. However, no such association was found for ARHGAP19 and MAP1B. These findings pinpoint ARHGAP27 and MAPT as potential susceptibility genes for ASD, with ARHGAP27(OR = 1.195, 95%CI:1.082–1.318. p<0.001) linked to an increased risk and MAPT(OR = 0.832, 95%CI:0.744–0.930, p<0.001) to a decreased risk of developing ASD.

### Summary findings

Drawing on insights from TWAS, WGCNA, MR, and GO, KEGG, and PPI networks, along with Conditional analysis, we have stratified genes implicated in ASD by their level of association confidence. ARHGAP27 emerges as a gene with robust associative evidence for ASD, corroborated by congruent findings spanning all aforementioned methodologies. Similarly, MAPT garners high confidence through consistent support from analyses encompassing TWAS, WGCNA, MR, along with GO, KEGG, PPI evaluations. In contrast, our assurance in the ASD linkage with ARHGAP19 and MAP1B is comparatively reserved; while these genes were identified through WGCNA, subsequent validation via MR did not reinforce their significance, intimating potential linkage disequilibrium (LD) or a pleiotropic scenario wherein both the expression quantitative trait loci (eQTLs) of ARHGAP19, MAP1B, and the SNP may concurrently influence the trait of social seclusion associated with ASD.

## Discussion

In this study, we integrated exploratory and confirmatory research methods, utilizing techniques such as TWAS, WGCNA, MR, PPI analysis, KEGG, and GO. Our goal was to reveal the transcriptional-level association between the ARHGAP and MAP gene families and ASD. Preliminary explorations with TWAS (ARHGAP27 TWAS.P = 6.62E-06, MAPT TWAS.P = 6.62E-06) and WGCNA (ARHGAP19, MAP1B) suggested potential associations of ARHGAP27, MAPT, ARHGAP19, and MAP1B with ASD. However, subsequent MR validation confirmed only the associations of ARHGAP27 and MAPT, with KEGG and GO (GTPase activator activity) analyses providing supportive evidence. PPI, core gene, and conditional analyses further validated the association of these two genes. Therefore, we are confident that differential expression of ARHGAP27 and MAPT in the brain is related to ASD risk: ARHGAP27 may increase risk, while MAPT seems to reduce it. TWAS analysis (Table 1) demonstrated statistical significance for ARHGAP27 and MAPT; WGCNA also identified relevant associations for ARHGAP19 and MAP1B as detailed in Figs 5 and 6. MR analysis (Table 2) yielded statistically significant results with P-values well below 0.01. Under these rigorous analytical conditions, we hold a high degree of confidence in our research findings.

Prior studies have indicated that ARHGAP and MAP gene families exert a notable influence on ASD [27, 28], potentially through the modulation of neuronal development, connectivity, and intracellular signaling pathways. Specifically, ARHGAP27 [29], which encodes for a Rho GTPase activating protein involved in cytoskeletal remodeling and cellular motility [9], has been implicated in various neurological conditions and anxiety disorders [29, 30]. ARHGAP27 is a gene that encodes for Rho GTPase-activating protein (RhoGAP) [31]. RhoGAPs are a class of proteins that regulate the activity of Rho GTPases. Rho GTPases are enzymes that play a significant role in various biological processes within the cell, such as dynamic changes in the cytoskeleton, cell movement, synapse formation, and neuronal connectivity [32]. The activity of Rho GTPases [33] can switch between an active state bound to GTP (guanosine triphosphate) and an inactive state bound to GDP (guanosine diphosphate) [34, 35]. RhoGAPs, including the protein encoded by ARHGAP27, can promote this switch from the GTP-bound active state to the GDP-bound inactive state [35], thus inhibiting the activity of Rho GTPases. In simpler terms, ARHGAP27 is a gene whose encoded RhoGAP protein can reduce the activity of Rho GTPases. Moreover, some studies have found variations in the expression of certain members of the Rho GTPase family in patients with ASD [36–38]. This suggests that Rho GTPase activity may play a role in the pathogenesis of ASD. However, further functional studies are needed to understand the role of ARHGAP and Rho GTPases in ASD.Conversely.MAPT [39] is the gene responsible for encoding the microtubule-associated protein Tau. Within the cell nucleus, the DNA sequence of the MAPT gene is transcribed into messenger RNA (mRNA). This mRNA is then transported to the cytoplasm where it undergoes translation on the ribosomes, leading to the assembly of a specific amino acid sequence that folds to form the Tau protein. The amino acid sequence of Tau protein determines its structure and function within the cell, especially its interaction with microtubules. Consequently, it plays a vital role in the stability and communication functions of neuronal cells. It is closely associated with the stabilization, organization, and structural regulation of microtubules [40]. Under normal conditions, the Tau protein [41] helps to maintain the stability of microtubules within cells, thus supporting the morphology and function of neurons. Therefore, current research indicates that in the general population, the regulation of Tau protein is positively correlated with the MAPT gene [42]. However, this positive correlation may be reversed in patients with ASD [43, 44]. Meanwhile, some studies have found that abnormal Tau protein aggregation may exist in a subset of ASD patients. These abnormal Tau accumulations may be related to the neuropathology and cognitive impairments of ASD [45]. In addition, genetic variations related to Tau protein have also been found to be associated with an increased risk of ASD [44], further suggesting the potential role of Tau protein in ASD. Furthermore, in our GO enrichment analysis, we discovered biological biochemical processes related to Tau protein associated with GTPase activator activity. All these are consistent with the causal effects between genes and ASD that we discovered in the MR validation stage (ARHGAP27 is related to an increased risk of developing ASD, while MAPT is related to a decreased risk of ASD).

The rigors of our dual discovery-validation approach, combined with the novel integration of TWAS and WGCNA, alongside MR for result verification [46], lend robustness and novelty to our findings. The selection of SNPs from the GTExv8 eQTL dataset under stringent criteria (*p<1e-5*) underscores the precision of our validation. Our multi-pronged analytical strategy minimizes method-specific errors and fosters confidence in identifying ASD-related genes.

Nevertheless, our study is subject to certain limitations, including the limited heritability of brain transcriptome data based on SNPs and potential biases reflecting human population diversity. Moreover, our conclusions are rooted in statistical analysis, with the genetic mechanisms underpinning ASD’s pathogenesis yet to be elucidated, necessitating further functional investigation.

## Conclusion

In conclusion, we have furnished a compendium of corroborative evidence that substantiates the causal linkage between ARHGAP27, MAPT, and ASD. Furthermore, we have presented substantial evidence of a significant genetic correlation between ARHGAP27 and ASD. Collectively, these insights contribute to unraveling the neurobiological underpinnings of ASD and underscore the necessity for continued investigative studies.

## Supporting information

S1 Table TWAS analysis results of all gene names identified as susceptibility genes associated with ASD. (DOCX)

S2 Table GO enrichment analysis. (DOCX)

S1 Checklist Human participants research checklist. (DOCX)
