# Supplementary material for: Identifying the impact of ARHGAP and MAP gene families on autism spectrum disorders
Source: PLoS One. 2024 Nov 8;19(11):e0306759. doi: 10.1371/journal.pone.0306759 (PMC11548836; doi:10.1371/journal.pone.0306759)
Supplement: S1 Table — (DOCX) [file pone.0306759.s001.docx]

Supplementary Table 1: TWAS Analysis Results of All Gene Names Identified as Susceptibility Genes Associated with ASD

| A2M | ANXA2R | BSN | CAMK1 |  | CHP1 | HPSE2 | LINC00307 | LOC101927653 | LTBP4 | NPC1L1 | PRR7 | RP11-495P10.6 | SLC29A4 | TMEM120B | ZNF254 |
| --- | --- | --- | --- | --- | --- | --- | --- | --- | --- | --- | --- | --- | --- | --- | --- |
| ABCB9 | AP000230.1 | BTG4 | CARTPT |  | CHPT1 | HSBP1L1 | LINC00317 | LOC101927703 | LTK | NPHP3-AS1 | PRRG1 | RP11-506E9.3 | SLC2A11 | TMEM150B | ZNF259P1 |
| ABCG2 | AP001605.4 | BTN2A3P | CASC16 |  | CHRNA10 | HSD11B2 | LINC00423 | LOC101927761 | LYL1 | NPHS2 | PRSS16 | RP11-508N22.12 | SLC2A12 | TMEM178B | ZNF442 |
| ABHD17B | AP1S1 | BVES | CATR1 |  | CHRNA4 | HSD3B1 | LINC00460 | LOC101927877 | LYPD1 | NPY1R | PRSS3P2 | RP11-53B2.2 | SLC31A2 | TMEM235 | ZNF446 |
| ABHD4 | APBA1 | C10orf126 | CBLN1 |  | CHRNA9 | HSDL1 | LINC00472 | LOC101927907 | LYPD3 | NRDE2 | PRSS54 | RP11-540O11.1 | SLC38A5 | TMEM255B | ZNF470 |
| AC005606.14 | APOBEC3F | C10orf90 | CC2D1A |  | CHRNB1 | HSPB6 | LINC00523 | LOC101927934 | LYPLA2 | NRG3 | PSG7 | RP11-63A11.1 | SLC41A2 | TMEM259 | ZNF514 |
| AC012065.7 | APOH | C11orf83 | CCDC11 |  | CHRNB3 | HTN1 | LINC00535 | LOC101928069 | LZTS3 | NRXN3 | PSMF1 | RP11-66N11.8 | SLC43A1 | TMEM26 | ZNF530 |
| AC013463.2 | AQP1 | C11orf86 | CCDC129 |  | CITED1 | HTR3C | LINC00560 | LOC101928134 | MAGEA6 | NTRK3 | PTCH2 | RP11-673E11.2 | SLC48A1 | TMEM37 | ZNF536 |
| ACKR1 | AQPEP | C12orf54 | CCDC137 |  | CITED4 | HUWE1 | LINC00588 | LOC101928173 | MAGEB4 | NTRK3-AS1 | PTGER3 | RP11-6F2.5 | SLC4A1 | TMEM95 | ZNF577 |
| ACMSD | ARHGAP19 | C14orf37 | CCDC153 |  | CLDN18 | HYPM | LINC00595 | LOC101928200 | MAGEE2 | NUDT10 | PTHLH | RP11-710C12.1 | SLC4A3 | TMOD1 | ZNF620 |
| ACOT7 | ARHGEF38 | C14orf79 | CCDC184 |  | CLEC11A | IDI2-AS1 | LINC00626 | LOC101928222 | MAGI2 | NUDT17 | PTK6 | RP11-774O3.3 | SLC4A5 | TNFAIP8L3 | ZNF628 |
| ACSM3 | ARL8A | C15orf41 | CCDC28B |  | CLEC4GP1 | IFI27L1 | LINC00630 | LOC101928269 | MAMLD1 | NUDT6 | PTPRA | RP11-873E20.1 | SLC51A | TNFSF11 | ZNF629 |
| ACTL6B | ARSI | C15orf62 | CCDC57 |  | CLEC4M | IFITM10 | LINC00638 | LOC101928283 | MAN2B2 | NXPE4 | PTPRS | RP11-893F2.14 | SLC5A7 | TNFSF18 | ZNF667 |
| ACTR3BP2 | ART3 | C16orf3 | CCDC58 |  | CLIC1 | IFNA5 | LINC00643 | LOC101928409 | MANEAL | NXPH1 | PTTG2 | RP11-90C4.2 | SLC5A8 | TNN | ZNF671 |
| ADAM12 | ART5 | C16orf95 | CCDC68 |  | CLIC5 | IGDCC4 | LINC00687 | LOC101928476 | MAP1B | OAZ1 | PURB | RP11-932O9.4 | SLC6A13 | TNNI3 | ZNF696 |
| ADAM18 | ASPG | C17orf104 | CCDC70 |  | CLSTN2-AS1 | IGF2-AS | LINC00865 | LOC101928647 | MAPK12 | OBP2B | PVRL4 | RP11-998D10.7 | SLC6A8 | TOM1L1 | ZNF70 |
| ADAMTSL1 | ATAD2B | C17orf53 | CCDC8 |  | CMKLR1 | IGFBP3 | LINC00900 | LOC101928661 | MAPRE3 | ODF1 | PWP2 | RP13-122B23.8 | SLC6A9 | TP53I3 | ZNF71 |
| ADAMTSL3 | ATF3 | C17orf59 | CCK |  | CNKSR1 | IGSF5 | LINC00919 | LOC101928710 | 45353 | OLFM3 | PXDC1 | RP3-333B15.4 | SLCO1A2 | TPBGL | ZNF717 |
| ADARB2 | ATP2A1 | C17orf78 | CCNO |  | CNPPD1 | IHH | LINC00923 | LOC101928751 | 45359 | ONECUT2 | PXDN | RP3-336K20__B.2 | SLCO1C1 | TPGS2 | ZNF79 |
| ADCK2 | ATP6V0A4 | C1QC | CCR8 |  | COA7 | IL3RA | LINC00924 | LOC101928767 | MARVELD2 | OPRK1 | PYCR1 | RP3-496C20.1 | SMARCD3 | TPRXL | ZNF836 |
| ADCY6 | ATP6V0C | C1orf177 | CCZ1 |  | COL12A1 | ILF3-AS1 | LINC00933 | LOC101928894 | MAST1 | OPRL1 | R3HCC1L | RP3-508D13.1 | SMG6 | TPTE | ZNF843 |
| ADM5 | AY927499 | C1orf213 | CD101 |  | COL1A1 | ING4 | LINC00948 | LOC101929034 | MATN3 | OPTC | R3HDM4 | RP3-522P13.2 | SMG7-AS1 | TRAF1 | ZRANB3 |
| ADPRHL1 | B4GALNT1 | C20orf78 | CD1B |  | COL22A1 | INSRR | LINC00957 | LOC101929078 | MBNL3 | OR10C1 | RAB11FIP4 | RP4-539M6.14 | SMIM2-AS1 | TRAK2 | ZSCAN31 |
| ADRB1 | BACE1 | C21orf2 | CD244 |  | COLCA2 | INTU | LINC00960 | LOC101929123 | MCM3 | OR1D5 | RAB3A | RP4-594L9.2 | SMIM24 | TRAP1 | ZSWIM5 |
| AEBP1 | BAI1 | C22orf46 | CDC34 |  | COPS7B | IQCA1 | LINC00963 | LOC101929133 | MED28 | OR1F1 | RAB3B | RP4-675G8.2 | SMR3A | TRDN | ZYG11A |
| AF070581 | BC024169 | C2CD4C | CDH22 |  | CORIN | IQCF5 | LINC01029 | LOC101929164 | MEIS3 | OR1F2P | RAB40A | RP4-742J24.2 | SNCG | TRIM17 |  |
| AGER | BC028044 | C2orf27A | CDH5 |  | CPT1A | IRX2 | LINC01056 | LOC101929167 | METRNL | OR1G1 | RAB4B | RP5-1154L15.2 | SNED1 | TRIM2 |  |
| AICDA | BC041998 | C2orf70 | CDK15 |  | CREB3L3 | ISG20L2 | LINC01197 | LOC101929180 | METTL21C | OR2C3 | RAB7B | RP9 | SNORA71B | TRNAU1AP |  |
| AIFM3 | BC042029 | C2orf72 | CDK2 |  | CREG1 | ISL1 | LINC01204 | LOC101929196 | MFSD4 | OR2F2 | RAMP2 | RPAIN | SNORD8 | TRPC4 |  |
| AK130486 | BC042374 | C6orf118 | CDKL2 |  | CRISPLD1 | ISM1-AS1 | LINC01222 | LOC101929210 | MGC15705 | OR5V1 | RARB | RPH3AL | SNRNP35 | TRPM2 |  |
| AKAP14 | BCAR3 | C6orf201 | CDT1 |  | CRX | ITGAD | LINC01315 | LOC101929260 | MGC15885 | OSBP2 | RARG | RPL3L | SNX1 | TRPM2-AS |  |
| AKAP6 | BEST4 | C8orf34 | CDYL2 |  | CRYBB3 | ITGB1BP2 | LINC01342 | LOC101929261 | MGC20647 | OSCP1 | RASA3 | RPP40 | SNX3 | TSIX |  |
| AL590762.11 | BFSP1 | C9orf135-AS1 | CEACAM21 |  | CRYM | ITGB4 | LINC01395 | LOC101929279 | MICAL2 | OVOL1 | RASSF8 | RPS6KA6 | SORCS1 | TSPAN5 |  |
| ALAS2 | BFSP2 | C9orf24 | CELSR3 |  | CSF1 | ITGB7 | LINC01432 | LOC101929289 | MIR31HG | OXER1 | RAVER2 | RRP1 | SORCS3-AS1 | TSPO2 |  |
| ALDH16A1 | BIRC5 | C9orf37 | CENPI |  | CSF2 | ITSN1 | LINC01442 | LOC101929325 | MIR4296 | OXGR1 | RBBP8NL | RSPH3 | SOX11 | TSSC4 |  |
| ALDH7A1 | BLACAT1 | CACFD1 | CERCAM |  | CSMD2 | JAZF1 | LIPE | LOC101929524 | MIR4313 | P2RX2 | RBFOX2 | RSPO2 | SOX7 | TTBK1 |  |
| ALKBH3-AS1 | BMP15 | CACNG1 | CERS3 |  | CSMD2-AS1 | KCND3 | LIPJ | LOC101929631 | MKRN1 | PACRG | RBM38 | RSPO3 | SPACA7 | TTLL11 |  |
| ALOX12P2 | BNIP3L | CACNG4 | CFHR2 |  | CST6 | KCNK12 | LIX1 | LOC101929681 | MKRN2 | PACSIN3 | RBPMS | RTKN | SPAG11A | TUB |  |
| AMN | BOD1L2 | CALCB | CFHR3 |  | CST9 | KCNK3 | LKAAEAR1 | LOC101929705 | MLIP | PADI1 | REG3G | RUNDC3A | SPAG5-AS1 | TUNAR |  |
| AMPH | BOLL | CALCR | CHDC2 |  | CTB-102L5.7 | KCNQ3 | LMOD3 | LOC101929762 | MLLT10 | PAEP | REG4 | S100A16 | SPATA21 | TXNDC2 |  |
| ANHX | BPIFC | CALHM1 | CHDH |  | CTC-384G19.1 | KCNS1 | LOC100126784 | LOC101930657 | MLX | PAGE5 | RETNLB | S100A5 | SPATA41 | TYR |  |
| ANKDD1A | BRE-AS1 | CALML4 | CHGA |  | CTD-2008P7.1 | KCNS3 | LOC100128993 | LOC102723542 | MMD2 | PARP2 | RFPL1 | SAP30BP | SPATA45 | UBAC2 |  |
| ANKLE1 | BRICD5 | CALN1 | CHIA |  | CTD-2537I9.16 | KCTD17 | LOC100129884 | LOC102723684 | MMP1 | PCDH18 | RGAG4 | SCGB2A1 | SPNS1 | UBE2D4 |  |
| ANKUB1 | BRINP1 | CALU | CHODL |  | CTD-2541J13.1 | KCTD4 | LOC100130078 | LOC102723847 | MMP24-AS1 | PCDHGC3 | RGMB-AS1 | SCGN | SPRR2B | UBE2DNL |  |
| DOC2B | F3 | FOXC2 | GRB7 |  | CTD-2561B21.11 | KHSRP | LOC100130370 | LOC102723864 | MNAT1 | PCGF5 | RGN | SCRG1 | SRRD | UBE2Q1 |  |
| DOCK9-AS2 | FAM104A | FOXK1 | GREM2 |  | CTD-2611O12.6 | KIAA0509 | LOC100130417 | LOC102724891 | MOK | PCSK1N | RGS5 | SDCCAG8 | SRSF5 | UBOX5 |  |
| DOPEY2 | FAM120AOS | FOXN3-AS1 | GRID1-AS1 |  | CTD-3193O13.1 | KIAA1522 | LOC100131262 | LOC102725383 | MORF4L2-AS1 | PDCD1LG2 | RGS6 | SEL1L2 | SSTR2 | UNC119B |  |
| DPEP1 | FAM124B | FOXP3 | GRIN2B |  | CUBN | KIAA1549L | LOC100131347 | LOC145678 | MPDZ | PDCD5 | RGSL1 | SELENBP1 | SSTR4 | USB1 |  |
| DQ576800 | FAM154A | FOXRED2 | GSK3A |  | CXCL12 | KIAA1614 | LOC100131508 | LOC145845 | MPZL2 | PDGFB | RHD | SEMA6C | ST8SIA6-AS1 | USH1G |  |
| DQ599616 | FAM167B | FSCN3 | GSN-AS1 |  | CXCR3 | KIAA1654 | LOC100131581 | LOC145945 | MRAP2 | PDYN | RHOD | SEPN1 | STAC3 | USP26 |  |
| DRD5 | FAM169B | FSD2 | GSPT1 |  | CXorf58 | KIAA2022 | LOC100132661 | LOC157740 | MRC2 | PDZD9 | RILP | 45543 | STC2 | USP49 |  |
| DSCAM | FAM186A | FSIP1 | GTPBP10 |  | CYLC1 | KIF20B | LOC100133920 | LOC158960 | MRGPRG-AS1 | PEX5L | RIMS1 | SERGEF | STIP1 | VAC14-AS1 |  |
| DSCAM-AS1 | FAM210B | FSTL4 | H1FOO |  | CYMP | KIF25 | LOC100287590 | LOC283177 | MRGPRX3 | PGC | RIN1 | SERPINA3 | STK19 | VCX2 |  |
| DSG4 | FAM212B-AS1 | FXYD2 | H2BFM |  | CYP2A6 | KIF2B | LOC100287877 | LOC283731 | MRM1 | PHAX | RIPPLY2 | SERPINB7 | STOX1 | VN1R1 |  |
| DST | FAM224A | FXYD4 | HABP2 |  | CYP2J2 | KIF5C | LOC100288181 | LOC284263 | MRPS26 | PHLDB1 | RMND5B | SFRP2 | STRADB | VN1R10P |  |
| DUOX1 | FAM46C | GABBR2 | HAGHL |  | CYP2S1 | KIR2DS1 | LOC100289495 | LOC284395 | MSRB3 | PIF1 | RNF10 | SFTA2 | STX6 | VSIG2 |  |
| DUSP15 | FAM50A | GABRA4 | HAO1 |  | CYP4Z2P | KIR2DS5 | LOC100294362 | LOC285095 | MUC5B | PIGR | RNF11B | SGCZ | STXBP5-AS1 | WDFY3-AS2 |  |
| DYNC1I1 | FAM65C | GABRG3 | HAO2 |  | CYR61 | KIRREL3-AS2 | LOC100505710 | LOC285857 | MVB12A | PIM1 | RNF122 | SGK2 | SUGT1P1 | WDFY4 |  |
| E2F2 | FAM71D | GALR3 | HAPLN4 |  | CYSLTR2 | KLB | LOC100505782 | LOC286058 | MXI1 | PINK1 | RNF123 | SH3GL1 | SULT1E1 | WDR45 |  |
| E2F4 | FAM81A | GAREM | HARS2 |  | DBF4B | KLF1 | LOC100506098 | LOC339685 | MXRA5 | PIP5KL1 | RNF126P1 | SH3GL1P1 | SWT1 | WDR46 |  |
| EFHB | FAM83G | GBGT1 | HBBP1 |  | DBH | KLHL25 | LOC100506272 | LOC401068 | MYOCD | PIPOX | RNF187 | SH3GL1P2 | SYPL2 | WDR62 |  |
| EFNA5 | FAM9A | GBX2 | HBD |  | DCAF12 | KLK8 | LOC100506411 | LOC401220 | MYOD1 | PITHD1 | RNF214 | SH3GLB2 | SYT4 | WDR65 |  |
| EIF1AD | FAM9B | GCNT3 | HBM |  | DCUN1D3 | KLRG2 | LOC100506797 | LOC440028 | MYT1L | PLCH1-AS1 | RORB | SHANK2 | SYT6 | WFDC2 |  |
| EIF4EBP1 | FBLN7 | GDA | HBQ1 |  | DDC-AS1 | KRBA1 | LOC100506834 | LOC441025 | NAA11 | PLXNA3 | RP1-155D22.2 | SHANK3 | TAAR3 | WFDC5 |  |
| ELK1 | FBP2 | GDF5 | HBZ |  | DDX4 | KRBA2 | LOC100506860 | LOC441204 | NAG18 | PLXNB2 | RP1-187B23.1 | SHE | TAC3 | WIPF3 |  |
| ELTD1 | FBRS | GHSR | HCCAT5 |  | DEFA4 | KRT1 | LOC100506895 | LOC441461 | NAPRT | PMP22 | RP11-1008C21.2 | SHISA7 | TANGO2 | WT1-AS |  |
| EMC9 | FBXL7 | GID4 | HDAC1 |  | DEFB121 | KRT14 | LOC100507140 | LOC553103 | NCAN | POLL | RP11-108K3.2 | SIGLEC6 | TBC1D21 | XRCC6 |  |
| EMILIN3 | FBXO40 | GIPC2 | HDAC11 |  | DEFB126 | KRT25 | LOC100507156 | LOC554207 | NCKIPSD | POLR2H | RP11-108P20.4 | SIRT3 | TBC1D25 | YBX2 |  |
| EMP1 | FBXO7 | GIT1 | HDGF |  | DENND2A | KRT36 | LOC100507165 | LOC57399 | NCS1 | POLR3E | RP11-109D9.4 | SIRT6 | TCEA3 | YBX3 |  |
| EPB42 | FBXO9 | GJC2 | HEPACAM2 |  | DGCR7 | KRT4 | LOC100507316 | LOC643085 | NDRG2 | POLR3K | RP11-115A15.2 | SKA1 | TCF21 | YPEL4 |  |
| EPHA7 | FCHO1 | GJD3 | HIF3A |  | DIO2 | KRT75 | LOC100507461 | LOC643711 | NDUFA9 | POM121L12 | RP11-1E4.1 | SLAMF9 | TDRD10 | ZBTB20-AS1 |  |
| EPHA8 | FECH | GKN1 | HILPDA |  | DISP2 | KRT79 | LOC100507501 | LOC647323 | NDUFAF5 | POM121L2 | RP11-217B1.2 | SLC13A3 | TEK | ZBTB39 |  |
| EPHB3 | FER1L5 | GLRX5 | HIRA |  | DLGAP1-AS2 | KRTAP4-12 | LOC100630923 | LOC653160 | NEGR1 | POM121L8P | RP11-21G15.1 | SLC14A1 | TELO2 | ZBTB8B |  |
| EPN2-AS1 | FEZ1 | GLUD2 | HIST1H4L |  | DLGAP2 | KRTAP5-9 | LOC100653005 | LOC728690 | NEU2 | PPAP2B | RP11-24D15.1 | SLC16A11 | TESC | ZC2HC1B |  |
| ERICH1 | FGD5 | GNA14 | HLF |  | DLK2 | KRTAP5-AS1 | LOC100996251 | LOC728868 | NEUROD6 | PPARG | RP11-266A24.1 | SLC16A4 | TEX13B | ZC3H12B |  |
| ERMP1 | FGF22 | GNAL | HMBS |  | DLST | LAS1L | LOC100996345 | LOC729173 | NEXN-AS1 | PPME1 | RP11-315F22.1 | SLC17A1 | TEX33 | ZCCHC3 |  |
| ESPN | FGF8 | GNAO1 | HMGCLL1 |  | DLX6-AS1 | LBX1 | LOC101060424 | LOC729870 | NFIA-AS2 | PPP1R12C | RP11-334J6.6 | SLC17A3 | TG | ZCCHC5 |  |
| ESPNL | FIP1L1 | GNG8 | HMGN2P46 |  | DMBT1 | LCE1E | LOC101926912 | LOC729970 | NFKB2 | PPP1R17 | RP11-338N10.1 | SLC17A6 | TGM2 | ZFAT |  |
| EU250746 | FLG-AS1 | GNPTG | HMGXB3 |  | DMTN | LDB3 | LOC101926996 | LOC730139 | NGF | PPP1R26-AS1 | RP11-359K18.3 | SLC17A9 | THNSL2 | ZFP1 |  |
| EXD2 | FLJ12825 | GOLM1 | HMP19 |  | DMWD | LECT1 | LOC101927038 | LONRF3 | NHSL2 | PPP1R2P9 | RP11-384P7.7 | SLC22A11 | THPO | ZFP92 |  |
| EXD3 | FLJ20021 | GPIHBP1 | HOMEZ |  | DNAJA4 | LEKR1 | LOC101927051 | LRRC16B | NKX2-5 | PPP1R3B | RP11-440L14.3 | SLC22A16 | THRA | ZFPM2 |  |
| EXO1 | FLJ30064 | GPR101 | HORMAD2 |  | DNAJC17 | LEO1 | LOC101927067 | LRRC2 | NNAT | PRAP1 | RP11-457K10.1 | SLC22A24 | THRB | ZFYVE19 |  |
| EXOC4 | FLJ32154 | GPR116 | HOXA6 |  | DNAJC5B | LGALS3 | LOC101927093 | LRRC3 | NOL10 | PRDX6 | RP11-461L18.1 | SLC22A31 | TIE1 | ZFYVE9 |  |
| EXOSC1 | FLJ35700 | GPR146 | HOXC10 |  | DNAL1 | LHX5 | LOC101927263 | LRRC37A5P | NOP14-AS1 | PREX2 | RP11-465B22.8 | SLC22A8 | TIMP4 | ZIC2 |  |
| EXTL3 | FLJ90680 | GPR26 | HOXD8 |  | DNASE1L3 | LILRB5 | LOC101927359 | LRRC42 | NOS1 | PRG2 | RP11-470M17.2 | SLC24A2 | TLK2 | ZMIZ1-AS1 |  |
| EZH1 | FLRT1 | GPR62 | HP09025 |  | DNMBP | LINC00210 | LOC101927507 | LRRC43 | NOVA2 | PRLHR | RP11-486G15.2 | SLC25A37 | TLR3 | ZMYM6 |  |
| F12 | FMO4 | GPRIN2 | HPCA |  | DNMT3L | LINC00244 | LOC101927550 | LRRC72 | NOX5 | PRMT7 | RP11-490G2.2 | SLC25A39 | TM4SF20 | ZMYND11 |  |
| F13B | FMO6P | GRASP | HPGDS |  | DNTT | LINC00302 | LOC101927616 | LSAMP | NPBWR2 | PRR15L | RP11-495K9.5 | SLC26A9 | TMEM105 | ZNF16 |  |
