# Supplementary material for: Identifying the impact of ARHGAP and MAP gene families on autism spectrum disorders
Source: PLoS One. 2024 Nov 8;19(11):e0306759. doi: 10.1371/journal.pone.0306759 (PMC11548836; doi:10.1371/journal.pone.0306759)
Supplement: S2 Table — (DOCX) [file pone.0306759.s002.docx]

Supplementary Table 2.GO Enrichment Analysis

| **Biological Process** | **CellComponnt** | **Biological Process** |
| --- | --- | --- |
| **establishment of mitochondrion localization,microtubule-mediated** | **main axon** | **SH3 domain binding** |
| **mitochondrion transport along microtubule** | **growth cone -** | **GTPase activator activity** |
| **establishment of mitochondrion localization** | **site of polarized growth** | **microtubule binding** |
| **positive regulation of microtubule polymerization.** | **dendritic spine** | **minor groove of adenine-thymine-rich DNA binding** |
| **positive regulation of microtubule polymerization or depolymerization** | **neurn spine** | **tubulin binding** |
| **positive regulation of axon extension** | **distal axon** | **dynactin binding** |
| **mitochondrion localization** | **axolemma** | **actin binding .** |
| **regulation of microtubule polymerization** | **microtubule** | **apolipoprotein binding** |
| **positive regulation of axonogenesis organelle transport along microtubule** | **apical dendrite** | **phospholipid binding** |
|  | **neuronal cell body** | **GTPase regulator activity -** |
