# Supplementary material for: Identifying the impact of ARHGAP and MAP gene families on autism spectrum disorders
Source: PLoS One. 2024 Nov 8;19(11):e0306759. doi: 10.1371/journal.pone.0306759 (PMC11548836; doi:10.1371/journal.pone.0306759)
Supplement: S1 Checklist — (DOCX) [file pone.0306759.s003.docx]

**Human Participants Research Checklist**

**Complete the following if your study involved human participants or human participants’ data. These questions should be addressed for prospective and retrospective studies.**

i. the day, month and year when the data were accessed for research purposes

Gene expression elucidates functional impact of polygenic risk for schizophrenia（doi: 10.1038/nn.4399.）2016 Nov

Characteristics and predictive value of blood transcriptome signature in males with autism spectrum disorders（doi: 10.1371/journal.pone.0049475.）2012

ASD,the GWAS data comes from the meta-analysis GWAS]at [ https://ww.med.unc.edu/pgc ] The data that support the findings of this study are openly 2017

available in [GTExv8 eQTL]at [(https://www.gtexportal.org/home/]. 2019

ii.whether authors had access to information that could identify individual participants during or after data collection

A
